# Likelihood of following a physician prescription to engage in the arts in the US

**DOI:** 10.1016/j.isci.2025.113877

**Published:** 2025-11-05

**Authors:** Daisy Fancourt, Randy Cohen, Jill Sonke

**Affiliations:** 1 Department of Behavioural Science and Health, UCL, London, UK; 2 Americans for the Arts, Washington, DC, USA; 3 Center for Arts in Medicine, University of Florida, Gainesville, FL, USA

**Keywords:** public health, neuroscience, behavioral neuroscience, psychology

## Abstract

Social prescribing of activities such as arts (“arts on prescription”) is growing globally, but it is unclear how much these prescriptions change behaviors. In data from the 2023 Americans Speak Out About the Arts Survey (*n* = 2,996 US adults), 76% said they were somewhat or very likely to follow a doctor’s recommendation if they were given a prescription to engage in the arts. The strongest predictors of positive responses were experience of past health benefits from the arts, belief in future health benefits, high educational attainment, approval of government arts funding, existing engagement in the arts, and being female. Applying behavior change frameworks, the strongest identified predictors relate to reflective motivations, suggesting that uptake may be increased via programs that explain what arts on prescription is, how it works, why patients are receiving referrals, what the evidence base is, and what benefits patients may experience.

## Highlights

• It is unclear whether arts prescriptions are likely to be taken up by recipients
• 76% of US adults say they are likely to follow a doctor’s advice to engage in the arts
• Strongest predictors include experiencing or believing in health benefits of the arts
• Improving reflective motivations to engage in arts may support arts prescribing

## Graphical abstract

**Figure:**
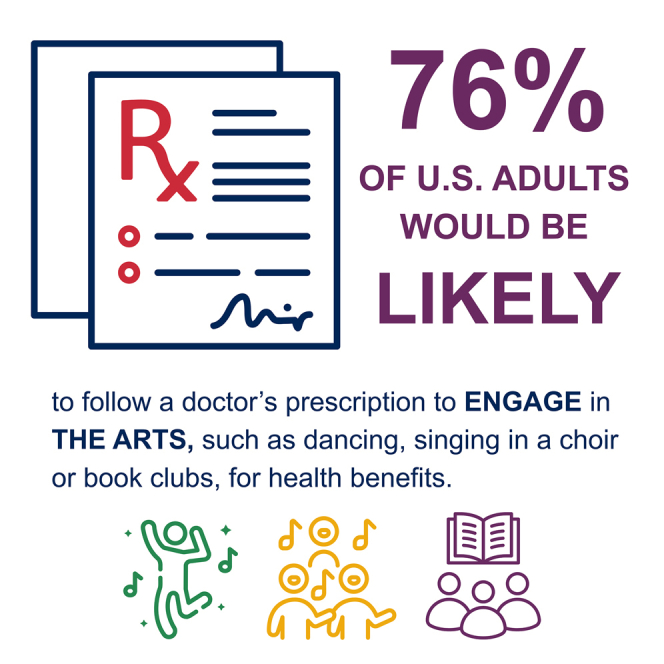

## Introduction

Healthcare systems around the world are demonstrating increasing engagement with social prescribing—a holistic, person-centered, and community-based approach that connects individuals to non-clinical support within the community.^1^ Social prescribing programs are now present in over 30 countries globally, ranging from small-scale regional pilots to full national programs.^2^ While social prescribing encompasses referrals to diverse organizations and interventions from nature-based projects, to physical activity, and broader advice and information resources, one of the most prominent types of referral is to arts-based activities (also known as “arts on prescription” or “arts prescribing”).

These referrals to arts and cultural activities reflect understanding that arts engagement is a valuable health behavior. Arts engagement encompasses different modes or “ways” of engaging in the arts (attending, creating, participating, consuming, and learning through) across different types of art forms or disciplines (including dance/movement, literary arts, media, music, theater/performance, and visual arts, crafts, and design).^3^ Theoretically, arts engagement is increasingly recognized as a health behavior that consists of diverse salutogenic active ingredients that activate psychological, biological, social, and behavioral mechanistic processes that lead to mental and physical health outcomes.^4^^,^^5^^,^^6^ In recent years, a number of randomized trials have demonstrated that clinical referral to arts programs can lead to tangible improvements in clinical outcomes.^7^^,^^8^ However, what remains less well understood is how people perceive receiving a referral to the arts from a clinician (i.e., an arts prescription), and, more specifically, whether arts prescriptions are likely to be taken up by recipients.

Work on social prescribing more broadly (i.e., encompassing activities including and beyond the arts) has demonstrated that care providers can find engaging patients with social prescribing difficult if there is not strong public understanding about programs or if there is skepticism around benefits and patients instead anticipate medical solutions.^9^ Notably, this barrier remains even in countries such as the UK where social prescribing has been in operation for several decades and is available as part of a national program.^10^ Skepticism among staff that patients are likely to attend activities once referred has also been highlighted as a barrier to staff engagement in social prescribing.^9^ Understanding who is likely to take up referrals is important from an equity perspective. Arts engagement in societies all around the world is unequal, with similar “social gradients” observed in participation in the arts as in health outcomes.^11^^,^^12^ It is, therefore, important that any programs that attempt to refer people to the arts are well designed and targeted not just to engage those individuals who are already likely to engage but also to engage those who would not otherwise engage. Notably, analyses of patterns of equity in uptake of social prescriptions in the UK as part of the national roll-out of social prescribing show that individuals facing socioeconomic hardship with greatest health needs are most likely to receive and take up prescriptions.^13^^,^^14^ However, whether this pattern is observed for arts-based prescriptions specifically, and in settings where arts on prescription or social prescribing programs more generally are not as widely used, remains unknown.

Consequently, this study sought to understand (1) whether people feel that they are likely to follow doctor referrals to engage in the arts as part of arts “prescriptions” and (2) what factors predict a perceived likelihood of following such referrals. We specifically focused on the context of the US, which is a unique context given the country has a highly medicalized concept of health and spends more on healthcare but has the lowest relative life expectancy and the highest rates of avoidable deaths among high-income countries globally.^13^ Although the US has developed health programs that consider issues such as food, housing, transportation, and employment over the past two decades, it is only in the last few years that broader social prescribing programs, including arts on prescription programs, have begun to emerge. As such, the US provides an opportunity to assess relatively early-stage attitudes to arts on prescription, with the potential both to track how they change and develop as pilots grow and expand, and also to develop targeted initiatives to ensure that arts on prescription programs can reach and engage individuals equitably.

## Results

### Sample characteristics

Data were taken from the *Americans Speak Out About the Arts in 2023 Survey* led by Americans for the Arts in the US; a survey conducted by Ipsos Public Affairs calibrated to be representative of the US population. Likelihood of accepting an arts prescription was asked in the survey with the question “If your doctor wrote you a prescription to participate in the arts as a means of improving your physical or mental health (singing in a choir, taking a ceramics class, dancing, or joining a book club, etc.), how likely are you to follow their recommendation?” Of the 3,062 adults in the survey, 2,996 provided complete data and were included in the analyses, 52.5% were female, 70.1% were White, and 54.6% had a degree qualification, showing close alignment with US Census statistics (Table 1). Overall, 51% said they had actively participated in the arts in the past year, but 76% said they were somewhat or very likely to follow a doctor’s recommendation if they were given a prescription to engage in the arts.

**Table 1:** Sample characteristics

	Likely to follow arts prescription			
	No/not very	Somewhat/very	Total	Pearson’s chi-square test
*N*	707 (23.6%)	2,289 (76.4%)	2,996 (100.0%)	
Sex				
Male	400 (56.6%)	1,022 (44.6%)	1,422 (47.5%)	<0.001
Female	307 (43.4%)	1,267 (55.4%)	1,574 (52.5%)	
Age generation				
Millennial (born 1982–2004)	172 (24.3%)	755 (33.0%)	927 (30.9%)	<0.001
Gen-X (born 1965–1981)	222 (31.4%)	729 (31.8%)	951 (31.7%)	
Baby Boomer (born 1946–1964)	217 (30.7%)	619 (27.0%)	836 (27.9%)	
Silent (born before 1946)	96 (13.6%)	186 (8.1%)	282 (9.4%)	
Race				
White	517 (73.1%)	1,582 (69.1%)	2,099 (70.1%)	0.130
Black	72 (10.2%)	279 (12.2%)	351 (11.7%)	
Hispanic	71 (10.0%)	283 (12.4%)	354 (11.8%)	
Other	47 (6.6%)	145 (6.3%)	192 (6.4%)	
Educational attainment				
Grade or high school	401 (56.7%)	959 (41.9%)	1,360 (45.4%)	<0.001
AA or BA degree	218 (30.8%)	950 (41.5%)	1,168 (39.0%)	
Postgraduate degree	88 (12.4%)	380 (16.6%)	468 (15.6%)	
Household income				
Under $50k	375 (53.0%)	986 (43.1%)	1,361 (45.4%)	<0.001
$50k–<$100k	228 (32.2%)	824 (36.0%)	1,052 (35.1%)	
$100k+	104 (14.7%)	479 (20.9%)	583 (19.5%)	
Employment status				
Employed	315 (44.6%)	1,260 (55.0%)	1,575 (52.6%)	<0.001
Retired/student/not working	274 (38.8%)	743 (32.5%)	1,017 (33.9%)	
Unemployed	118 (16.7%)	286 (12.5%)	404 (13.5%)	
Marital status				
Single	225 (31.8%)	697 (30.4%)	922 (30.8%)	0.018
Married/domestic partnership	327 (46.3%)	1,182 (51.6%)	1,509 (50.4%)	
Widowed/separated/divorced	155 (21.9%)	410 (17.9%)	565 (18.9%)	
Living with children				
No	575 (81.3%)	1,641 (71.7%)	2,216 (74.0%)	<0.001
Yes	132 (18.7%)	648 (28.3%)	780 (26.0%)	
Urbanicity				
Rural	184 (26.0%)	477 (20.8%)	661 (22.1%)	0.001
Suburban	358 (50.6%)	1,145 (50.0%)	1,503 (50.2%)	
Urban	165 (23.3%)	667 (29.1%)	832 (27.8%)	
Political party				
Republican	319 (45.1%)	812 (35.5%)	1,131 (37.8%)	<0.001
Democrat	228 (32.2%)	1,085 (47.4%)	1,313 (43.8%)	
Other	160 (22.6%)	392 (17.1%)	552 (18.4%)	
Engaged in arts activities				
No	519 (73.4%)	962 (42.0%)	1,481 (49.4%)	<0.001
Yes	188 (26.6%)	1,327 (58.0%)	1,515 (50.6%)	
Engaged in cultural activities (in person)				
No	333 (47.1%)	357 (15.6%)	690 (23.0%)	<0.001
Yes	374 (52.9%)	1,932 (84.4%)	2,306 (77.0%)	
Engaged in cultural activities (online)				
No	496 (70.2%)	999 (43.6%)	1,495 (49.9%)	<0.001
Yes	211 (29.8%)	1,290 (56.4%)	1,501 (50.1%)	
Personal importance of arts				
No	387 (54.7%)	344 (15.0%)	731 (24.4%)	<0.001
Yes	320 (45.3%)	1,945 (85.0%)	2,265 (75.6%)	
Belief in health benefits				
No	427 (60.4%)	474 (20.7%)	901 (30.1%)	<0.001
Yes	280 (39.6%)	1,815 (79.3%)	2,095 (69.9%)	
Experience of health benefits				
No	566 (80.1%)	812 (35.5%)	1,378 (46.0%)	<0.001
Yes	141 (19.9%)	1,477 (64.5%)	1,618 (54.0%)	
Approve of arts funding				
No	480 (67.9%)	669 (29.2%)	1,149 (38.4%)	<0.001
Yes	227 (32.1%)	1,620 (70.8%)	1,847 (61.6%)	
Approval of arts-health funding				
No	311 (44.0%)	363 (15.9%)	674 (22.5%)	<0.001
Yes	396 (56.0%)	1,926 (84.1%)	2,322 (77.5%)	

*N* = 2,996.

**Figure 1:**
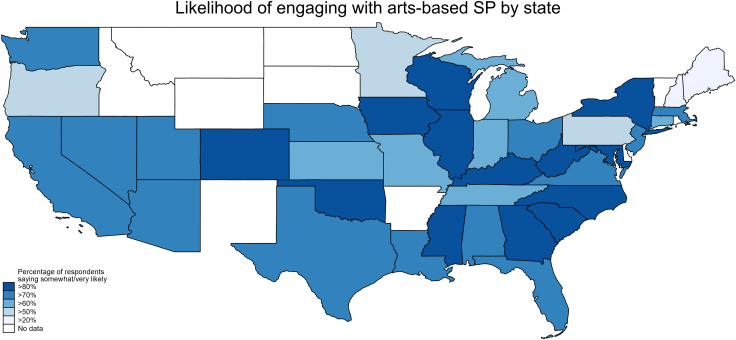
Reported likelihood of following an arts-based prescription if referred by a doctor by US state (weighted)

When viewed by state, weighted statistics showed that there was higher reported likelihood of taking up an arts-based prescription in the Midwest and Southeast than in the West or Southwest (Figure 1).

**Figure 2:**
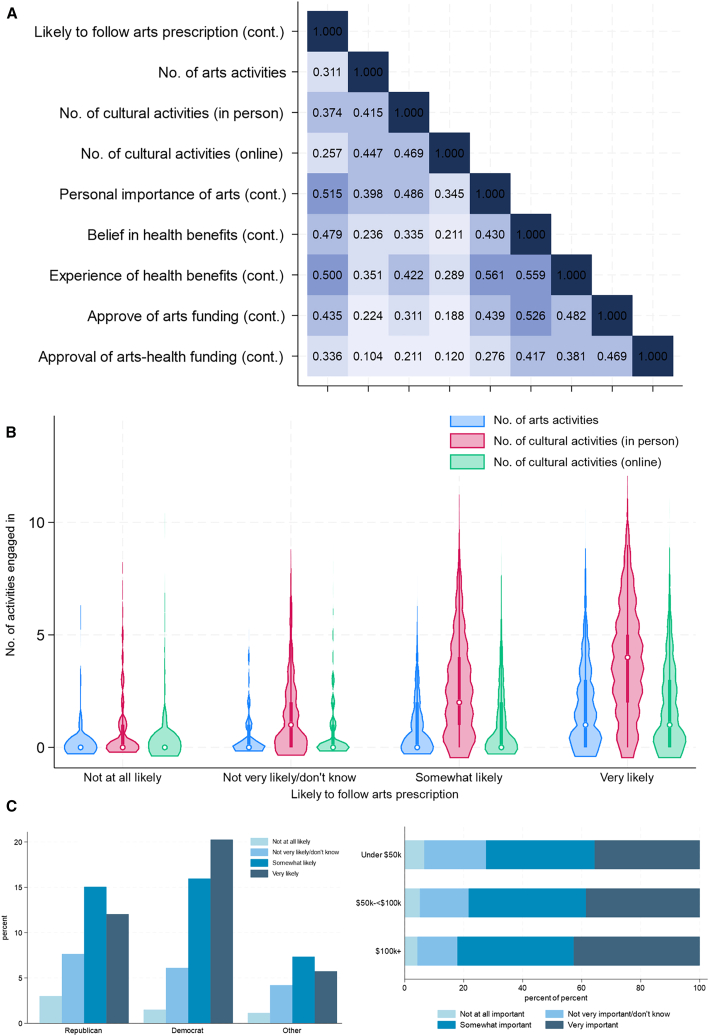
Descriptive figures of outcome and predictors (A) Heat plot of correlations between arts-related variables. (B) Violin plots of likelihood of following an arts prescription by number of arts and cultural activities already engaged in. (C) Bar charts of likelihood of following an arts prescription by political affiliation and household income.

Reported likelihood of following an arts prescription was correlated with all measures of arts and cultural participation in the past year and attitudes and beliefs around the arts. Most prominently, past experience of health and wellbeing benefits of engaging in the arts, personal importance of the arts within one’s life and belief about the capability of the arts to improve future health and well-being were most strongly correlated, explaining 23%–27% of the variance in overall likelihood to follow an arts prescription (Figure 2A). People who engaged more frequently in arts and culture responded most positively to whether they would follow an arts prescription (Figure 2B), as did people with higher household income and those who reported political democratic identification (Figure 2C).

### Regression analyses

#### Demographic predictors

Multiple logistic regression models were used to explore likelihood of taking up an arts-on-prescription referral. Women were 1.6 times more likely to report being likely to follow an arts prescription than men (OR 1.55, 95% CI 1.17–2.05). Compared to Millennials, every previous generation reported a lower likelihood of following a referral (37% lower likelihood for Gen-X, 44% lower for Baby Boomers, and 56% lower for Silent generations). There was no difference by race (Table S1).

#### Socioeconomic predictors

Those with higher educational attainment reported higher likelihoods of following an arts prescription (2.9 times higher for those with a postgraduate degree—95% CI 1.74–4.69—and 1.9 times higher for those with an AA or BA degree compared to those without degrees—95% CI 1.31–2.63). However, there was no difference by household income or employment status.

#### Social predictors

Neither marital status, living with children, nor urbanicity predicted reported likelihood of following an arts prescription, but those who reported identifying politically with Democrats were 2.4 times more likely to believe they would follow an arts prescription compared to independents (95% CI 1.63–3.52). Republicans were neither more nor less likely than independents to believe they would follow an arts prescription (OR 1.11, 95% CI 0.76–1.62).

#### Arts predictors

Arts engagement in the past year was a strong predictor of likelihood to follow an arts prescription. Those who had participated in arts activities (i.e., actively making, performing, practicing, or sharing creative arts, culture, or crafts activities) were 2.8 times more likely to believe they would follow an arts prescription than those who had not participated (95% CI 1.94–3.95). Similarly, those who had engaged in cultural activities (i.e., going to watch, visit or see arts performed or exhibited by others) were 2.7 times more likely (in person engagement; 95% CI 1.89–3.87) and 2.1 times more likely (online engagement; 95% CI 1.44–2.97) to believe they would follow an arts prescription compared to those who had not engaged in cultural activities in the past year.

#### Arts values

People who believed the arts were important in their lives were 2.3 times more likely to believe they would follow an arts prescription (95% CI 1.64–3.28). Those who had previous experience of health and well-being benefits of the arts were 3.4 times more likely (95% CI 2.23–5.10), while those who believed in health benefits (even if they had not experienced them personally) were 2.5 times more likely (95% CI 1.72–3.65). Those who approved of government funding to arts organizations were 1.9 times more likely to believe they would follow an arts prescription (95% CI 1.30–2.75), and those who specifically approved of funding for arts-health programs were marginally more likely (OR 1.46, 95% CI 0.99–2.13).

#### Overall

**Figure 3:**
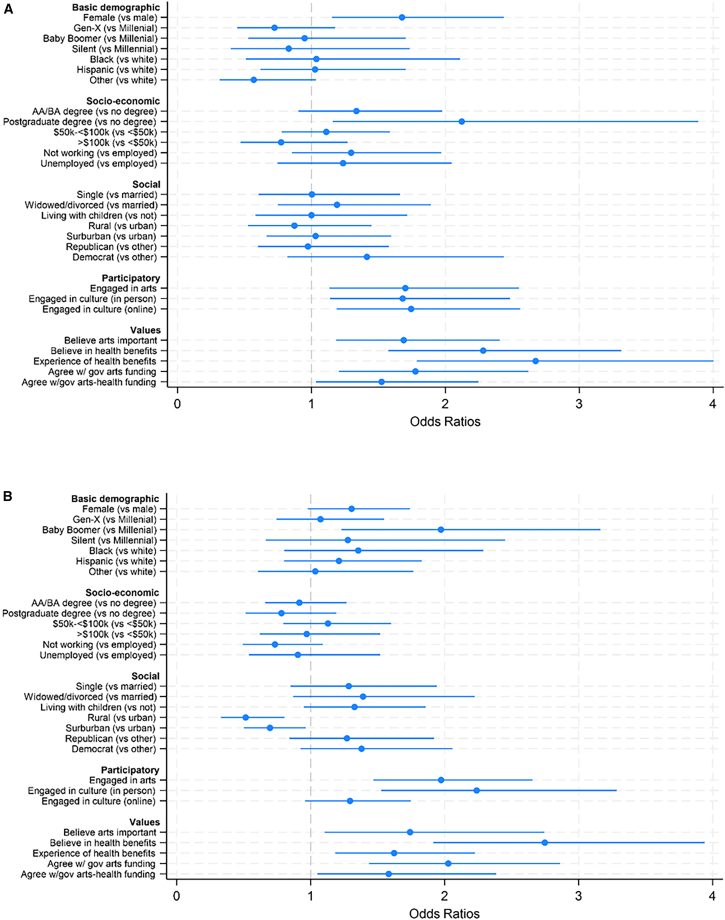
Predictors of reporting being likely to follow an arts prescription if referred by a doctor (weighted, with 95% confidence intervals) (A) Somewhat/very likely. (B) Very likely only.

Fully adjusted models showed that the strongest predictors of reporting being likely to follow an arts prescription (in descending order) were experience of past health benefits from the arts (OR 2.67, 95% CI 1.79–4.00), belief in future health benefits (OR 2.28, 95% CI 1.57–3.31), high educational attainment (OR 2.12, 95% CI 1.16–3.89), approval of government arts funding (OR 1.78, 95% CI 1.21–2.62), existing engagement in the arts (OR 1.70, 95% CI 1.13–2.55), and being female (OR 1.68, 95% CI 1.15–2.44) (Figure 3 and Table S1).

When just using “very likely to engage” as the outcome, results were maintained, although those in rural and suburban settings were less likely to believe they would follow the referral (49% and 30% less likely, respectively), and online participation and educational attainment no longer predicted engagement. Instead, belief in the health benefits of engaging in the arts became an even stronger predictor (OR 2.74 95% CI 1.92–3.94; see Figure 3).

## Discussion

Over three-quarters of adults in the US report that they believe they will follow recommendations to engage in the arts if they are written a prescription by their doctor. This is a major finding given that only half of adults in the sample reported having actively participated in arts activities in the past year, suggesting that arts on prescription could help to increase national rates of arts participation; an important target given increasing recognition that arts participation is a health behavior with diverse mental and physical health benefits.^15^^,^^16^ Notably, there is no evidence that those who are more marginalized are less likely to take up prescriptions (such as those with lower household income, who are unemployed, or living alone). However, there is a clear pattern of greater perceived likelihood of following an arts prescription among adults who are younger, more educated, more politically liberal, and who are already engaged in and value the arts, which follows predictors of arts engagement generally. This is important to identify and acknowledge, as it suggests that in the absence of greater efforts to explain arts-based prescriptions, those who actually follow recommendations to engage may present a skewed sample. There are also initial suggestions that arts prescriptions may be better received in some geographical regions of the country, such as the Midwest and Southeast, which could support place-based approaches to developing arts prescribing schemes further.

Applying behavior change theory, these findings provide insights into potential enablers of following arts prescriptions, with clear implications for future endeavors to develop equitable arts on prescription programs, systems, and policy within the US. The finding of higher reported likelihood of following a prescription among those with higher educational attainment and past experience of and belief in benefits suggests that strong reflective motivations to engage could be an enabler of prescription uptake.^17^ Reflective motivation can be achieved through increasing knowledge and understanding and eliciting positive feelings about behavioral target, such as through providing communications and marketing materials outlining the health benefits of the arts.^17^ Such resources may be important to provide to individuals at the point arts prescriptions are discussed by a healthcare professional to provide a stronger rationale and incentive to engage. Indeed, a national study of pilot arts on prescription programs in 23 locations around the US completed in 2024 highlighted that a fundamental reoccurring barrier related to limited awareness of what social prescribing is,^18^ demonstrating the importance of addressing this barrier. Work on social prescribing more broadly has also highlighted challenges around developing a shared understanding of what social prescribing services and pathways should look like, hindering staff in being able to describe social prescribing effectively to patients, and consequently adversely affecting referrals.^9^ However, social prescribing programs that highlight the long-term benefits of engagement (presenting evidence showing that arts or other social prescribing activities as more than short-term solutions) have had greater success at engaging and retaining participants.^19^

Similarly, the strong predictive effects of past engagement in the arts and values relating to the arts (such as approval of arts funding and belief that the arts are important to individuals), as well as the relationship with liberal values, suggest that automatic motivations could also be key enablers. Automatic motivation can be achieved through imitative learning and habit formation, such as through providing easy, free opportunities for arts engagement within communities.^17^ For individuals who may be more reluctant to engage with arts prescriptions, more organic opportunities to sample arts activities prior to formal clinician recommendations (such as through taster sessions run in conjunction with healthcare settings) may remove barriers such as individuals not believing they are “artistic.” The patterning of likelihood by generation suggests that social opportunities could also be an important enabler, which could be leveraged to improve uptake of arts-based social prescribing through encouraging (1) intergenerational arts activities that provide behavioral modeling from younger generations more likely to engage, (2) providing greater variety in the kinds of arts activities provided on prescription to ensure that individuals from all cultural and socioeconomic backgrounds have offers of activities that resonate with them and their personal backgrounds, and (3) ensuring that any physical barriers to participation (e.g., use of spaces that are not fully accessible) are addressed.^17^ Indeed, it is notable that when looking at predictors of being very likely to engage, living in nonurban locations was a barrier, and online participation in cultural activities became nonsignificant, suggesting that physical opportunities to engage may affect even people’s behavioral *intentions*.

### Limitations of the study

This analysis has many strengths, including its use of a large sample and bespoke survey questions that were designed to capture rich detail on individuals’ attitudes to and engagement with the arts. Questions were designed in conjunction with *Americans for the Arts’* Equity Task Force; participant focus groups; and a diversity, equity, inclusion, and accessibility consultant to incorporate appropriate breadth and cultural nuance. While the sampling methods were developed to provide a sample with similar characteristics to the US general population and used weights to align the sample to the national census, the lack of a full random sampling approach and internet-based method may have excluded individuals who may be most vulnerable. As such, this study does not provide insight into how arts-based social prescribing is likely to be received among those who arguably are most in need, and future studies specifically focusing on these populations are encouraged. Additionally, we focused on individuals’ beliefs about their behavioral intentions, rather than on the behaviors themselves. Intentions explain around 23% of the variance in health behaviors.^20^ However, other factors, such as capacity, autonomy, and descriptive norms also predict the behavior itself, and they were not explored in this study. Nonetheless, the findings presented here lay the foundation for future intervention studies that can explore more the dynamic interplay between intentions to follow arts-based social prescribing referrals and practical experiences of barriers and enablers. As another limitation, analyses are limited by the questions available in the survey. There was only one question about arts on prescription within the survey, limiting opportunities to understand attitudes to and previous engagement with the service more broadly. The survey did not include any measures of the health or well-being of participants, including whether they had any mental or physical health conditions that might have made them eligible for arts on prescription programs such as those that are currently being piloted across the US. It also did not ask for specificities about people’s health insurance or use of Medicaid or Medicare. Potential concerns about whether participation in arts on prescription could incur individual costs may have influenced findings, although it is notable that household income and employment status (which could have increased likelihood of having health insurance) did not predict responses. We were also only able to consider broad categories of race due to insufficient statistical power to examine differences by further subgroups. So, for future iterations of the *Americans Speak Out About the Arts* survey, it is, therefore, recommended that more comprehensive question batteries on social prescribing, health, and health insurance are included, as well as repeat questions on attitudes to arts on prescription in order to track which factors *change* behavioral intentions over time and over-sampling to enable deeper considerations of potential differences by race. Additional questions exploring other kinds of barriers and enablers (e.g., access to public transport, disabilities, leisure time, etc.) would also provide even greater scope to understand patterning in responses and develop targeted intervention recommendations. Further, while the sample for this study was recruited by Ipsos and calibrated to be representative of the US population, so it did *not* consist of people who were positively biased toward the arts, the questions on arts prescribing occurred in the context of questions focused on arts engagement more broadly, so this could have influenced how people approached their response. Integration of social prescribing questions within broader studies is thus encouraged in the future to corroborate findings. Our analyses did not look at interactions between predictors, as we did not have any prior data to make *a priori* hypotheses. However, based on our findings, we propose that future studies test specifically whether there are intersectional inequalities among the sociodemographic groups we identified as being less likely to say they would follow a referral, namely individuals of middle-to-older age, from minoritized ethnic groups, with lower levels of educational attainment. We also recommend exploring further the nexus of education and belief in health benefits, to understand further the extent to which reflective motivations are driven by cumulative years of education vs. more specific types of education (such as training in health sciences).

Overall, this study makes an important contribution by demonstrating that clinicians prescribing arts to patients could help to engage more people with arts activities, with 75% of sampled adults in the US saying they would be likely to follow a GP recommendation; a 50% increase on the number who reported engaging of their own accord in the last year. However, it highlights inequities in who believes they are most likely to follow arts prescriptions if they are referred that, if not addressed, could lead to inequities not just in engagement but in health benefits that may occur as a result of engagement. One of the core guiding principles for successful implementation of social prescribing at the embedding stage is creating awareness for addressing the wider determinants of health and the role social prescribing services can play.^21^ The strongest identified predictors of perceived likelihood of following a doctor referral to the arts relate to reflective motivations, suggesting that rich programs aimed at explaining what arts on prescription is, how it works, why patients are receiving referrals, what the evidence base is, and what benefits patients may experience could be fundamental to increasing uptake as pilot schemes of arts on prescription continue to develop in the US.

## Resource availability

### Lead contact

Further information and requests for resources should be directed to and will be fulfilled by the lead contact, Prof. Daisy Fancourt (d.fancourt@ucl.ac.uk).

### Materials availability

This study did not generate any new materials.

### Data and code availability

• Data are available from Americans for the Arts upon request. The website for the survey is listed in the key resources table.
• Original code has been deposited on OSF and is publicly available. DOIs are listed in the key resources table.
• Any additional information required to reanalyze the data reported in this paper is available from the lead contact upon request.

## Acknowledgments

The EpiArts Lab, a National Endowment for the Arts Research Lab at the 10.13039/100007698University of Florida, is supported in part by an award from the National Endowment for the Arts (1936473-38-24). The opinions expressed are those of the authors and do not represent the views of the National Endowment for the Arts Office of Research & Analysis or the National Endowment for the Arts. The National Endowment for the Arts does not guarantee the accuracy or completeness of the information included in this material and is not responsible for any consequences of its use. The EpiArts Lab is also supported by Americans for the Arts, 10.13039/100015283Bloomberg Philanthropies (F024567), the Dharma Endowment Foundation, the Pabst Steinmetz Foundation, and the State of Florida Division of Arts and Culture (24.c.ne.900.834).

## Author contributions

D.F., R.C., and J.S. conceived the study. R.C. coordinated data collection. D.F. analyzed the data and drafted the manuscript. All authors edited and approved the manuscript.

## Declaration of interests

J.K.S. is a guest editor for the special issue “Transdisciplinary approaches to arts and health: Integrating creative practice in clinical and public health contexts” but was not involved in any parts of the editorial handling of this article.

## STAR★Methods

### Key resources table

**Table:** 

REAGENT or RESOURCE	SOURCE	IDENTIFIER
**Other**		

Americans Speak Out About the Arts in 2023 Survey	Americans for the Arts	https://www.americansforthearts.org/by-program/reports-and-data/research-studies-publications/public-opinion-poll

**Software and algorithms**		

Statistical code	Open Science Forum	https://osf.io/wv3df/

### Method details

#### Dataset

Data were taken from the *Americans Speak Out About the Arts in 2023 Survey* led by Americans for the Arts in the US. The study was designed to gauge the public’s (1) level of personal engagement in the arts as both audience and creator, (2) support for arts education and government funding of arts and culture, and (3) opinions on the personal and well-being benefits that come from engaging in the arts. The survey was conducted from 5-11 July 2023 by Ipsos Public Affairs. A sample of 3,062 adults aged 18+ from the continental U.S., Alaska, and Hawaii were recruited and interviewed online in English. The sample was randomly drawn from Ipsos’ online panel, partner online panel sources, and “river” sampling (recruiting participants while they are engaged in other online activities). Respondent characteristics were calibrated to be representative of the U.S. Population using raking-ratio adjustments, using the target population of the US 2022 Census American Community Survey data. Post-hoc weights were made to the population characteristics on gender, age, race/ethnicity, region, education, and political party identification. Ipsos calculates a design effect (DEFF) for each study based on the variation of the weights.^22^ This study had a credibility interval adjusted for design effect of the following (n=3,062, DEFF=1.5 adjusted Confidence Interval=+/-3.7 percentage points) and a credibility interval of plus or minus 2.2 percentage points for all respondents.

For this analysis, we used data from this survey; no new data were collected. This study has Institutional Review Board approval from the University of Florida (IRB201901792) and ethical approval from University College London Research Ethics Committee (project 18,839/001). Due to small samples in Alaska and Hawaii, we focused on adults living within the continental US (n=3,022). Twenty-six participants were missing data on sex, so were excluded. Complete data were available for all remaining participants, providing a final analytical sample size of 2,996.

#### Outcome

Likelihood of accepting an arts prescription was asked with the question “If your doctor wrote you a prescription to participate in the arts as a means of improving your physical or mental health (singing in a choir, taking a ceramics class, dancing, or joining a book club, etc.), how likely are you to follow their recommendation?” Responses were on a five-point scale: very likely, somewhat likely, not very likely, not at all likely, don’t know. For descriptive statistics, the variable was recoded as both a four-point categorical variable (with “don’t know” reclassified as “not very likely”). However, as our research question focused on understanding the likelihood that individuals would follow doctor referrals to engage in the arts, we were specifically interested in the positive outcomes: reportedly being “very likely” or “somewhat likely” to follow recommendations. So for regression analyses we used a binary variable (“very/somewhat likely” vs other responses) and tested the consistency of findings in sensitivity analyses focusing specifically on “very likely” vs other responses.

#### Predictors

Demographic predictors included sex (male, female), age by generation (Millennial (born 1982-2004), Gen-X (born 1965-81), Baby Boomer (born 1946-64) and Silent (born before 1946)), and ethnicity (White, Black, Hispanic and other).

Socio-economic predictors included educational attainment (high school or less, Associate’s or Bachelor’s degree, and post-graduate degree), household income (under $50,000, $50,000-$100,000, and ≥$100,000) and employment status (employed (part or full time), retired/student/homemaker/not working, and unemployed).

Social predictors included marital status (single, married/domestic partnership, and widowed/separated/divorced), whether respondents were living with any children under the age of 18, urbanicity (rural, suburban, and urban), and which political party respondents reported most identifying with (republican, democrat or other).

Arts participation was assessed with three questions. First, respondents were asked whether, in the past year, they had visited, attended or watched any of the following in person: concert or musical performance (pop, gospel, classical, hip hop etc), museum (such as art, history, children's, or science), theatre performance, visual arts/crafts/craft show/art gallery, fairs or festivals (art, music, film, etc.), zoo/aquarium/botanical garden, media arts (artwork using technology, film and video, animation, robotics, etc.), historic site, poetry or literary event, dance performance, opera/musical theatre, or other. Next respondents were asked if they had done any of the same list of activities virtually/online. Finally, participants were asked if they had been personally involved in making, performing, practicing, or sharing creative arts, culture, or crafts activities—either at home or in the community, including painting, sculpting, taking creative photographs, doing ceramics, quilting or sewing, creating virtual content such as social media creations, videos or podcasts, making handmade objects (crafts, decorative art, jewelry, woodworking, or blacksmithing, etc.), designing (fashion, floral, home and interior, web, graphic, etc.), writing or reading poetry, dancing, acting or participating in theatre, singing with a group or in a choir, playing a musical instrument, or other. For each of these three variables, we created binary variables (any activity in the past year vs none) and count variables (how many of the activities people reported doing).

Values and attitudes to the arts were asked with five questions. Respondents were asked whether they felt the arts and culture were important to them personally (not at all, not very, somewhat and very). Their belief in the health benefits of the arts was assessed through averaging responses on whether they agreed with three statements (five-point Likert scale from strongly disagree to strongly agree): “the arts improve personal healing and the healthcare experience”, “the arts help people deal with mental health issues such as loneliness, isolation, depression, and anxiety” and “the arts assist some people in their recovery from substance abuse and addiction”. Experience of the health benefits of the arts was assessed through asking participants if “arts and culture has a positive effect on my overall health and well-being” and if “the arts have helped me cope during times of mental or emotional distress”, with participants assigned a score as to whether they agreed with none, one or both statements. Approval of arts funding was assessed through averaging responses on a five-point Likert scale (from strongly approve to strongly disapprove) on whether respondents approved or disapproved of the government investing in nonprofit arts and culture organisations when provided by local, state or federal government. Finally, approval of funding arts and culture to support health was assessed through asking respondents if they favoured or opposed funding arts and culture to address the issues of healthcare and healing, or mental health, with participants assigned a score as to whether they favoured none, one or both statements.

### Quantification and statistical analysis

Differences between those who were likely vs not likely to follow recommendations to engage in arts if referred by a doctor were calculated using Pearson’s Chi-square test. A map of reported likelihood by state was created using Stata’s *spmap* command, using ESRI shapefiles from the Environmental Systems Research Institute. Numbers of observations per state varied from 3 to 238. To reduce the influence of small cell sizes for demographic variables, we dropped states with fewer than 10 observations from the map (although these data were still included in subsequent statistical analyses). Data for each state were weighted using post-hoc weights (as detailed above).

Multiple logistic regression models were used to explore likelihood of taking up an arts-on-prescription referral. To avoid potential “Table 2 fallacy”, predictors were entered in blocks of related factors, each just adjusted for basic demographics.^23^ Model 1 was adjusted for demographic predictors and model 2-5 = model 1 + each category of predictors detailed above in turn (socio-economic, social, arts participation and values). However, model 6, which included all predictors together, is still presented for comparative purposes. All analyses were weighted using post-hoc weights (as detailed above). Main analyses binarised the outcome as “very/somewhat likely” vs other responses, while sensitivity analyses tested the stability of findings when using “very likely” vs other outcomes.

For regression models, we confirmed independence of observations through a scatterplot of the residuals. We confirmed no extreme outliers using Cook’s distance for each observation. Lastly, the absence of multicollinearity was verified with Variance Inflation Factor (VIF) values all below 10. Analyses were carried out in Stata v18.
