# Supplementary material for: Likelihood of following a physician prescription to engage in the arts in the US
Source: iScience. 2025 Nov 5;28(12):113877. doi: 10.1016/j.isci.2025.113877 (PMC12670935; doi:10.1016/j.isci.2025.113877)
Supplement: Document S1. Table S1 [file mmc1.pdf]

**iScience, Volume *28***

## Supplemental information

# Likelihood of following a physician prescription to engage in the arts in the US

**Daisy Fancourt, Randy Cohen, and Jill Sonke**

# Supplementary Material

Supplementary Table 1: Logistic Regression Model for Arts-based SP (would follow referral); odds ratios and confidence intervals

| | | 1 | 2 | 3 | 4 | 5 | 6 |
|---|---|---|---|---|---|---|---|
| Sex | Female | 1.55 [1.17, 2.05] ** | 1.66 [1.24, 2.22] *** | 1.56 [1.16, 2.08] ** | 1.91 [1.39, 2.62] *** | 1.43 [1.03, 1.99] * | 1.68 [1.15, 2.44] ** |
| Age generation | Gen-X (Born 1965-1981) | 0.63 [0.43, 0.90] * | 0.54 [0.37, 0.79] ** | 0.57 [0.39, 0.83] ** | 0.75 [0.50, 1.13] | 0.66 [0.43, 1.01] | 0.72 [0.45, 1.18] |
| | Baby Boomer (Born 1946-1964) | 0.56 [0.38, 0.83] ** | 0.49 [0.33, 0.75] *** | 0.58 [0.36, 0.93] * | 1.01 [0.65, 1.54] | 0.79 [0.50, 1.25] | 0.95 [0.53, 1.70] |
| | Silent (Born before 1946) | 0.44 [0.28, 0.68] *** | 0.35 [0.21, 0.58] *** | 0.42 [0.24, 0.71] ** | 0.92 [0.55, 1.53] | 0.80 [0.46, 1.39] | 0.83 [0.40, 1.73] |
| Race | Black | 0.93 [0.59, 1.46] | 1.02 [0.64, 1.62] | 0.77 [0.47, 1.25] | 0.87 [0.51, 1.47] | 1.23 [0.65, 2.35] | 1.04 [0.51, 2.11] |
| | Hispanic | 1.03 [0.66, 1.62] | 1.10 [0.70, 1.75] | 0.91 [0.58, 1.43] | 0.83 [0.50, 1.38] | 1.29 [0.79, 2.11] | 1.03 [0.62, 1.70] |
| | Other | 0.56 [0.32, 1.00] | 0.49 [0.27, 0.89] * | 0.55 [0.30, 0.98] * | 0.52 [0.29, 0.92] * | 0.64 [0.35, 1.17] | 0.57 [0.31, 1.03] |
| Educational attainment | AA or BA degree | | 1.86 [1.31, 2.63] *** | | | | 1.34 [0.90, 1.98] |
| | Postgraduate degree | | 2.86 [1.74, 4.69] *** | | | | 2.12 [1.16, 3.89] * |
| Household income | \$50k-<\$100k | | 1.11 [0.83, 1.49] | | | | 1.11 [0.78, 1.58] |
| | \$100k+ | | 1.07 [0.71, 1.62] | | | | 0.77 [0.47, 1.27] |
| Employment status | Retired/student/not working | | 1.01 [0.70, 1.47] | | | | 1.30 [0.85, 1.97] |
| | Unemployed | | 0.94 [0.63, 1.38] | | | | 1.24 [0.75, 2.05] |
| Marital status | Single | | | 0.84 [0.58, 1.24] | | | 1.00 [0.61, 1.66] |
| | Widowed/separated/divorced | | | 1.12 [0.76, 1.65] | | | 1.19 [0.75, 1.89] |
| Living with children | Yes | | | 1.27 [0.84, 1.92] | | | 1.00 [0.58, 1.71] |
| Urbanicity | Rural | | | 0.83 [0.53, 1.29] | | | 0.87 [0.53, 1.45] |
| | Surburban | | | 0.95 [0.66, 1.36] | | | 1.03 [0.67, 1.60] |
| Political party | Republican | | | 1.11 [0.76, 1.62] | | | 0.97 [0.60, 1.58] |
| | Democrat | | | 2.40 [1.63, 3.52] *** | | | 1.41 [0.82, 2.44] |
| Engaged in arts activities | Yes | | | | 2.77 [1.94, 3.95] *** | | 1.70 [1.13, 2.55] * |
| Engaged in cultural activities (in person) | Yes | | | | 2.70 [1.89, 3.87] *** | | 1.68 [1.14, 2.48] ** |
| Engaged in cultural activities (online) | Yes | | | | 2.07 [1.44, 2.97] *** | | 1.74 [1.19, 2.56] ** |
| Personal importance of arts | Yes | | | | | 2.32 [1.64, 3.28] *** | 1.69 [1.19, 2.41] ** |
| Belief in health benefits | Yes | | | | | 2.51 [1.72, 3.65] *** | 2.28 [1.57, 3.31] *** |
| Experience of health benefits | Yes | | | | | 3.37 [2.23, 5.10] *** | 2.67 [1.79, 4.00] *** |
| Approve of arts funding | Yes | | | | | 1.89 [1.30, 2.75] *** | 1.78 [1.21, 2.62] ** |
| Approval of arts-health funding | Yes | | | | | 1.46 [0.99, 2.13] | 1.52 [1.03, 2.25] * |
| Intercept | | 3.77 [2.70, 5.26] *** | 2.78 [1.85, 4.18] *** | 3.08 [1.70, 5.57] *** | 0.63 [0.39, 1.02] | 0.34 [0.22, 0.54] *** | 0.14 [0.06, 0.36] *** |
| Pseudo $R^2$ | | 0.02 | 0.05 | 0.05 | 0.15 | 0.25 | 0.29 |
| Wald $\chi^2$ | | 32.83, p<.001 | 68.81, p<.001 | 71.25, p<.001 | 161.30, p<.001 | 257.21, p<.001 | 367.70, p<.001 |
| Number of observations | | 2996 | 2996 | 2996 | 2996 | 2996 | 2996 |

**** p<.001, ** p<.01, * p<.05      N=2,996      Reference categories: Male; Millennials (Born 1982-2004); White*
